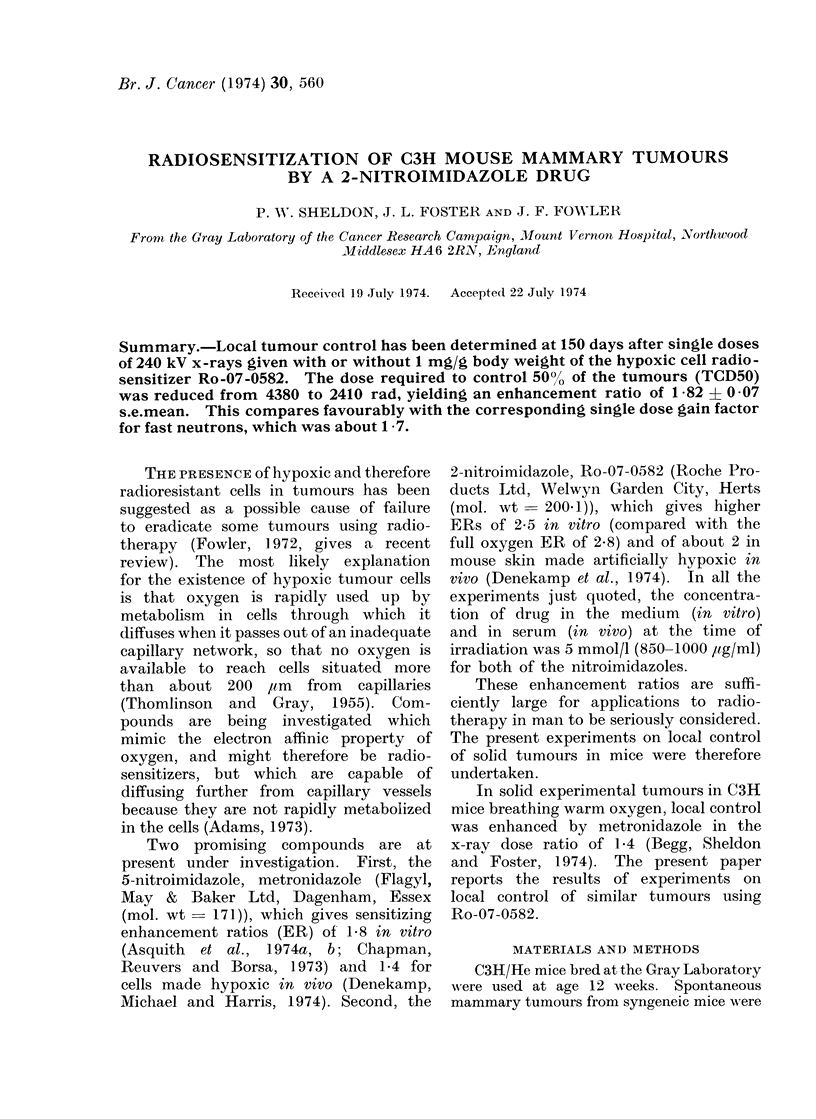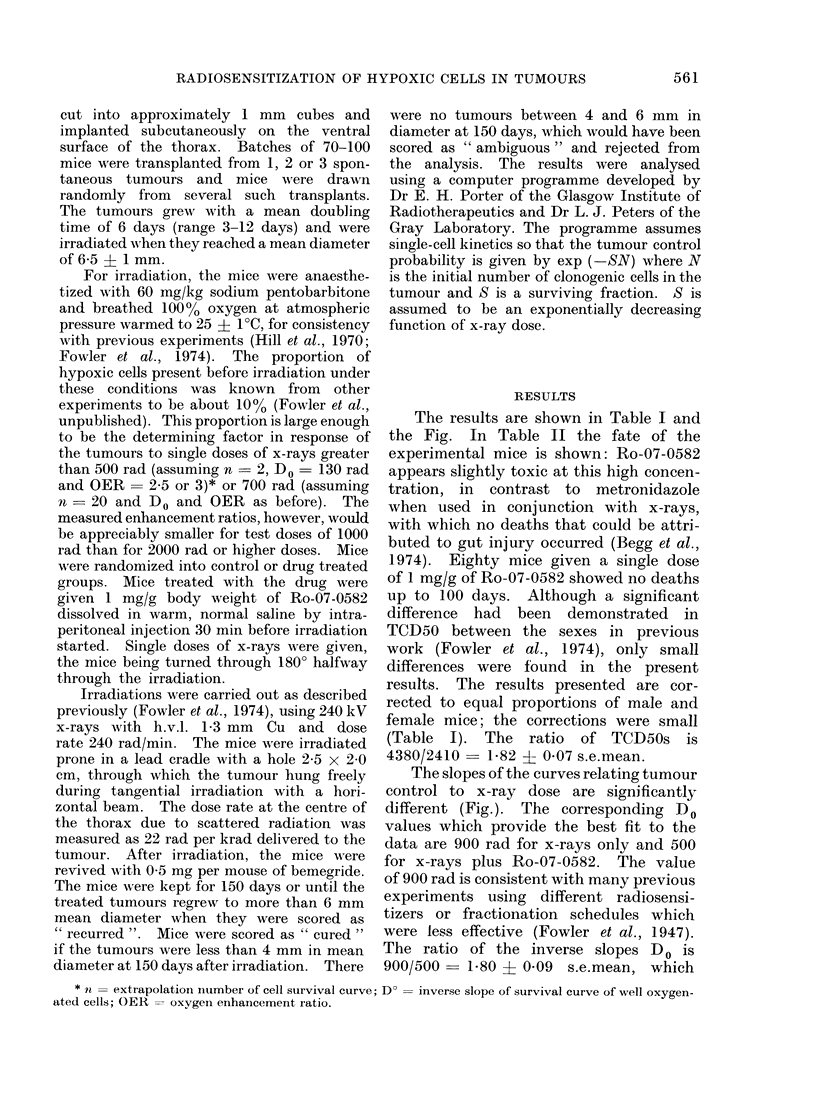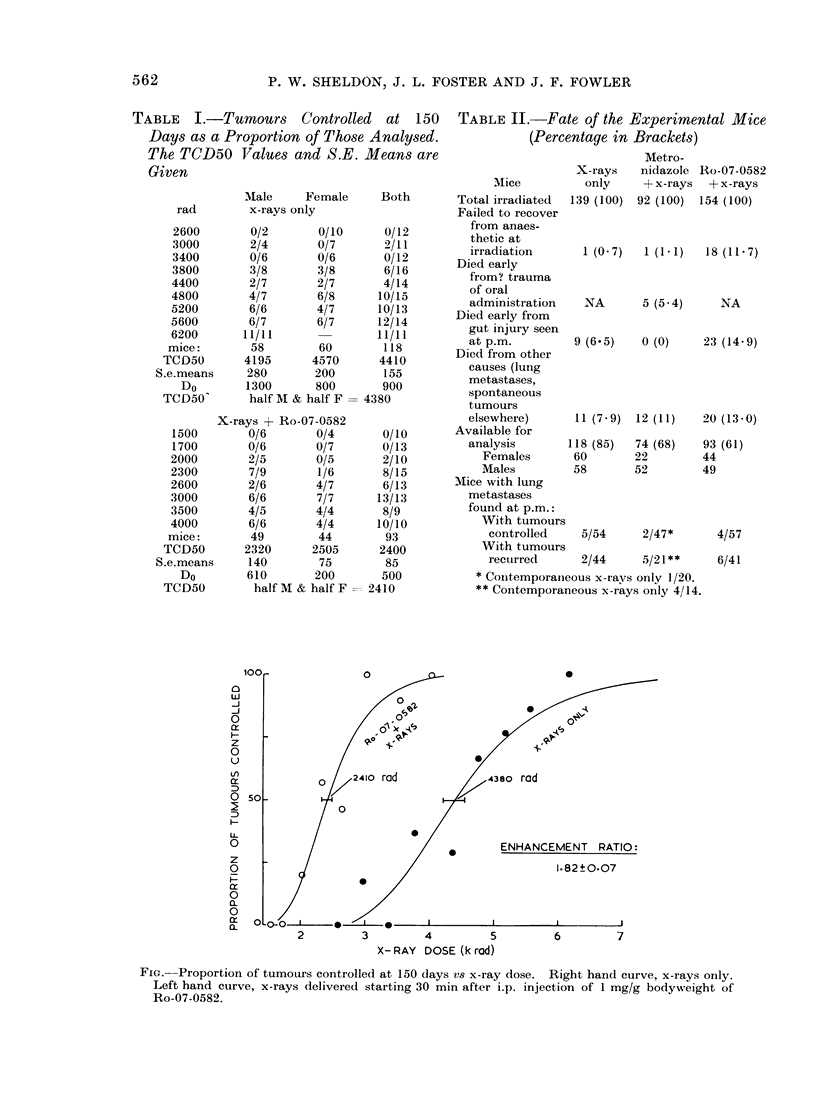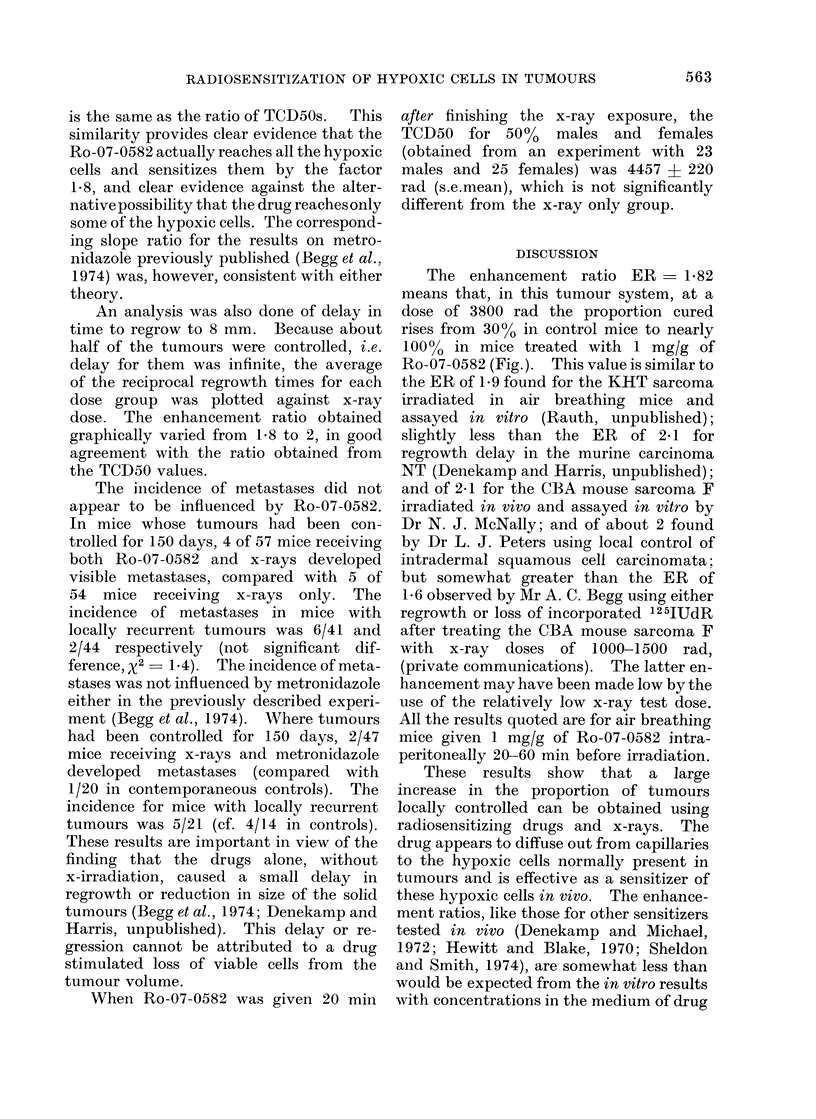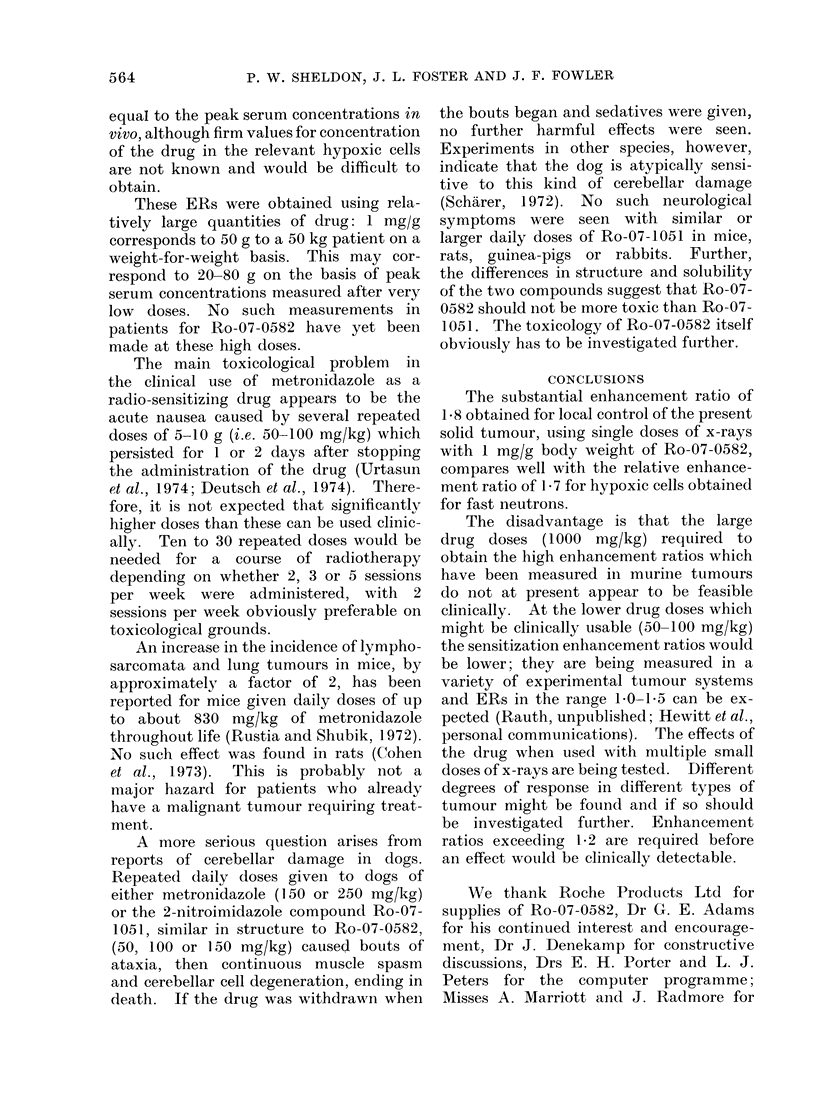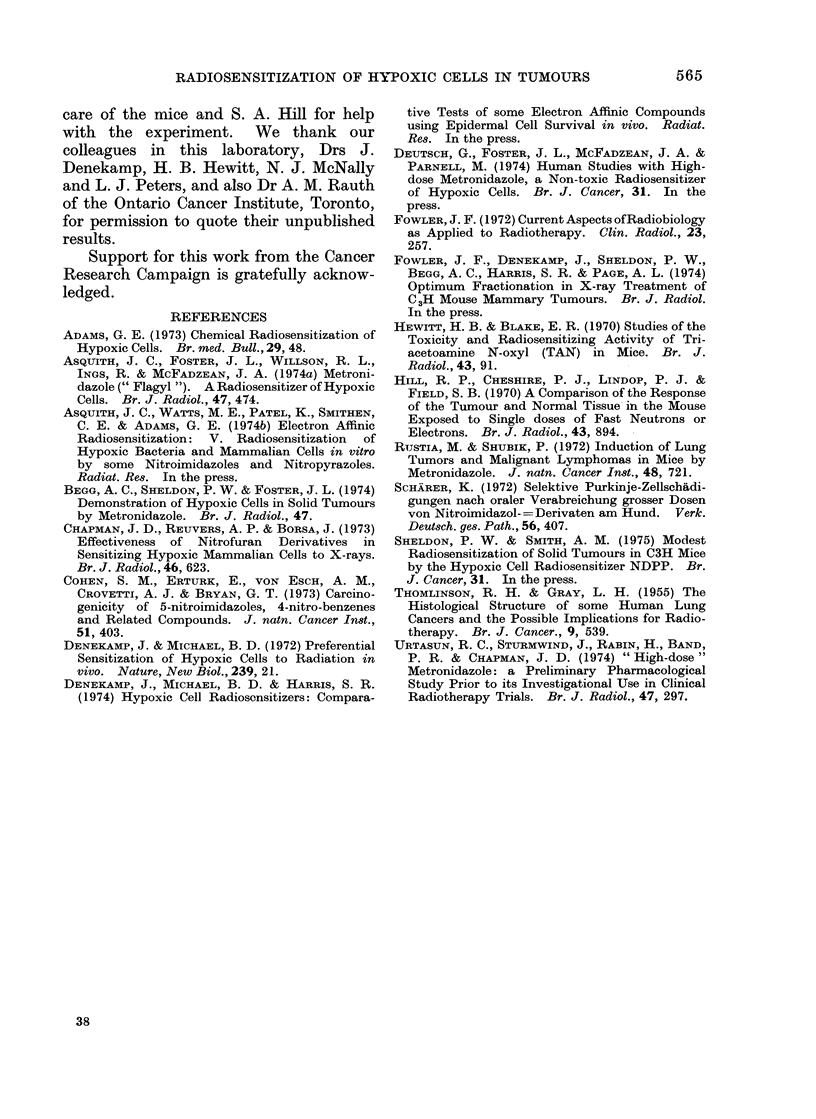# Radiosensitization of C3H Mouse Mammary Tumours by a 2-Nitroimidazole Drug

**DOI:** 10.1038/bjc.1974.235

**Published:** 1974-12

**Authors:** P. W. Sheldon, J. L. Foster, J. F. Fowler

## Abstract

Local tumour control has been determined at 150 days after single doses of 240 kV x-rays given with or without 1 mg/g body weight of the hypoxic cell radiosensitizer Ro-07-0582. The dose required to control 50% of the tumours (TCD50) was reduced from 4380 to 2410 rad, yielding an enhancement ratio of 1·82 ± 0·07 s.e.mean. This compares favourably with the corresponding single dose gain factor for fast neutrons, which was about 1·7.


*Br. J. Cancer* (1974) **30**, 560

### RADIOSENSITIZATION OF C3H MOUSE MAMMARY TUMOURS BY A 2-NITROIMIDAZOLE DRUG

P. W. SHELDON, J. L. FOSTER AND J. F. FOWLER

*From the Gray Laboratory of the Cancer Research Campaign, Mount Vernon Hospital, Northwood Middlesex HA6 2RN, England*

Received 19 July 1974. Accepted 22 July 1974

**Summary.—Local tumour control has been determined at 150 days after single doses of 240 kV x-rays given with or without 1 mg/g body weight of the hypoxic cell radiosensitizer Ro-07-0582. The dose required to control 50% of the tumours (TCD50) was reduced from 4380 to 2410 rad, yielding an enhancement ratio of 1·82 ± 0·07 s.e.mean. This compares favourably with the corresponding single dose gain factor for fast neutrons, which was about 1·7.**

THE PRESENCE of hypoxic and therefore radioresistant cells in tumours has been suggested as a possible cause of failure to eradicate some tumours using radiotherapy (Fowler, 1972, gives a recent review). The most likely explanation for the existence of hypoxic tumour cells is that oxygen is rapidly used up by metabolism in cells through which it diffuses when it passes out of an inadequate capillary network, so that no oxygen is available to reach cells situated more than about 200 μm from capillaries (Thomlinson and Gray, 1955). Compounds are being investigated which mimic the electron affinic property of oxygen, and might therefore be radiosensitizers, but which are capable of diffusing further from capillary vessels because they are not rapidly metabolized in the cells (Adams, 1973).

Two promising compounds are at present under investigation. First, the 5-nitroimidazole, metronidazole (Flagyl, May & Baker Ltd, Dagenham, Essex (mol. wt = 171)), which gives sensitizing enhancement ratios (ER) of 1·8 *in vitro* (Asquith *et al.*, 1974*a*, *b*; Chapman, Reuvers and Borsa, 1973) and 1·4 for cells made hypoxic *in vivo* (Denekamp, Michael and Harris, 1974). Second, the 2-nitroimidazole, Ro-07-0582 (Roche Products Ltd, Welwyn Garden City, Herts (mol. wt = 200·1)), which gives higher ERs of 2·5 *in vitro* (compared with the full oxygen ER of 2·8) and of about 2 in mouse skin made artificially hypoxic *in vivo* (Denekamp *et al.*, 1974). In all the experiments just quoted, the concentration of drug in the medium (*in vitro*) and in serum (*in vivo*) at the time of irradiation was 5 mmol/l (850–1000 μg/ml) for both of the nitroimidazoles.

These enhancement ratios are sufficiently large for applications to radiotherapy in man to be seriously considered. The present experiments on local control of solid tumours in mice were therefore undertaken.

In solid experimental tumours in C3H mice breathing warm oxygen, local control was enhanced by metronidazole in the x-ray dose ratio of 1·4 (Begg, Sheldon and Foster, 1974). The present paper reports the results of experiments on local control of similar tumours using Ro-07-0582.

### MATERIALS AND METHODS

C3H/He mice bred at the Gray Laboratory were used at age 12 weeks. Spontaneous mammary tumours from syngeneic mice were cut into approximately 1 mm cubes and implanted subcutaneously on the ventral surface of the thorax. Batches of 70–100 mice were transplanted from 1, 2 or 3 spontaneous tumours and mice were drawn randomly from several such transplants. The tumours grew with a mean doubling time of 6 days (range 3–12 days) and were irradiated when they reached a mean diameter of $6 \cdot 5 \pm 1$ mm.

For irradiation, the mice were anaesthetized with 60 mg/kg sodium pentobarbitone and breathed 100% oxygen at atmospheric pressure warmed to $25 \pm 1$°C, for consistency with previous experiments (Hill *et al.*, 1970; Fowler *et al.*, 1974). The proportion of hypoxic cells present before irradiation under these conditions was known from other experiments to be about 10% (Fowler *et al.*, unpublished). This proportion is large enough to be the determining factor in response of the tumours to single doses of x-rays greater than 500 rad (assuming $n = 2$, $D_0 = 130$ rad and OER = 2·5 or 3)* or 700 rad (assuming $n = 20$ and $D_0$ and OER as before). The measured enhancement ratios, however, would be appreciably smaller for test doses of 1000 rad than for 2000 rad or higher doses. Mice were randomized into control or drug treated groups. Mice treated with the drug were given 1 mg/g body weight of Ro-07-0582 dissolved in warm, normal saline by intraperitoneal injection 30 min before irradiation started. Single doses of x-rays were given, the mice being turned through 180° halfway through the irradiation.

Irradiations were carried out as described previously (Fowler *et al.*, 1974), using 240 kV x-rays with h.v.l. 1·3 mm Cu and dose rate 240 rad/min. The mice were irradiated prone in a lead cradle with a hole $2 \cdot 5 \times 2 \cdot 0$ cm, through which the tumour hung freely during tangential irradiation with a horizontal beam. The dose rate at the centre of the thorax due to scattered radiation was measured as 22 rad per krad delivered to the tumour. After irradiation, the mice were revived with 0·5 mg per mouse of bemegride. The mice were kept for 150 days or until the treated tumours regrew to more than 6 mm mean diameter when they were scored as " recurred ". Mice were scored as " cured " if the tumours were less than 4 mm in mean diameter at 150 days after irradiation. There were no tumours between 4 and 6 mm in diameter at 150 days, which would have been scored as " ambiguous " and rejected from the analysis. The results were analysed using a computer programme developed by Dr E. H. Porter of the Glasgow Institute of Radiotherapeutics and Dr L. J. Peters of the Gray Laboratory. The programme assumes single-cell kinetics so that the tumour control probability is given by exp $(-SN)$ where $N$ is the initial number of clonogenic cells in the tumour and $S$ is a surviving fraction. $S$ is assumed to be an exponentially decreasing function of x-ray dose.

### RESULTS

The results are shown in Table I and the Fig. In Table II the fate of the experimental mice is shown: Ro-07-0582 appears slightly toxic at this high concentration, in contrast to metronidazole when used in conjunction with x-rays, with which no deaths that could be attributed to gut injury occurred (Begg *et al.*, 1974). Eighty mice given a single dose of 1 mg/g of Ro-07-0582 showed no deaths up to 100 days. Although a significant difference had been demonstrated in TCD50 between the sexes in previous work (Fowler *et al.*, 1974), only small differences were found in the present results. The results presented are corrected to equal proportions of male and female mice; the corrections were small (Table I). The ratio of TCD50s is $4380/2410 = 1 \cdot 82 \pm 0 \cdot 07$ s.e.mean.

The slopes of the curves relating tumour control to x-ray dose are significantly different (Fig.). The corresponding $D_0$ values which provide the best fit to the data are 900 rad for x-rays only and 500 for x-rays plus Ro-07-0582. The value of 900 rad is consistent with many previous experiments using different radiosensitizers or fractionation schedules which were less effective (Fowler *et al.*, 1947). The ratio of the inverse slopes $D_0$ is $900/500 = 1 \cdot 80 \pm 0 \cdot 09$ s.e.mean, which

* $n$ = extrapolation number of cell survival curve; D° = inverse slope of survival curve of well oxygenated cells; OER = oxygen enhancement ratio.

TABLE I.—*Tumours Controlled at 150 Days as a Proportion of Those Analysed. The TCD50 Values and S.E. Means are Given*

| rad | Male | Female | Both |
|---|---|---|---|
| | x-rays only | | |
| 2600 | 0/2 | 0/10 | 0/12 |
| 3000 | 2/4 | 0/7 | 2/11 |
| 3400 | 0/6 | 0/6 | 0/12 |
| 3800 | 3/8 | 3/8 | 6/16 |
| 4400 | 2/7 | 2/7 | 4/14 |
| 4800 | 4/7 | 6/8 | 10/15 |
| 5200 | 6/6 | 4/7 | 10/13 |
| 5600 | 6/7 | 6/7 | 12/14 |
| 6200 | 11/11 | — | 11/11 |
| mice: | 58 | 60 | 118 |
| TCD50 | 4195 | 4570 | 4410 |
| S.e.means | 280 | 200 | 155 |
| $D_0$ | 1300 | 800 | 900 |
| TCD50` | half M & half F = 4380 | | |
| | X-rays + Ro-07-0582 | | |
| 1500 | 0/6 | 0/4 | 0/10 |
| 1700 | 0/6 | 0/7 | 0/13 |
| 2000 | 2/5 | 0/5 | 2/10 |
| 2300 | 7/9 | 1/6 | 8/15 |
| 2600 | 2/6 | 4/7 | 6/13 |
| 3000 | 6/6 | 7/7 | 13/13 |
| 3500 | 4/5 | 4/4 | 8/9 |
| 4000 | 6/6 | 4/4 | 10/10 |
| mice: | 49 | 44 | 93 |
| TCD50 | 2320 | 2505 | 2400 |
| S.e.means | 140 | 75 | 85 |
| $D_0$ | 610 | 200 | 500 |
| TCD50 | half M & half F = 2410 | | |

TABLE II.—*Fate of the Experimental Mice (Percentage in Brackets)*

| Mice | X-rays only | Metro-nidazole +x-rays | Ro-07-0582 +x-rays |
|---|---|---|---|
| Total irradiated | 139 (100) | 92 (100) | 154 (100) |
| Failed to recover from anaesthetic at irradiation | 1 (0·7) | 1 (1·1) | 18 (11·7) |
| Died early from? trauma of oral administration | NA | 5 (5·4) | NA |
| Died early from gut injury seen at p.m. | 9 (6·5) | 0 (0) | 23 (14·9) |
| Died from other causes (lung metastases, spontaneous tumours elsewhere) | 11 (7·9) | 12 (11) | 20 (13·0) |
| Available for analysis | 118 (85) | 74 (68) | 93 (61) |
| Females | 60 | 22 | 44 |
| Males | 58 | 52 | 49 |
| Mice with lung metastases found at p.m.: | | | |
| With tumours controlled | 5/54 | 2/47* | 4/57 |
| With tumours recurred | 2/44 | 5/21** | 6/41 |

* Contemporaneous x-rays only 1/20.

** Contemporaneous x-rays only 4/14.

FIG.—Proportion of tumours controlled at 150 days *vs* x-ray dose. Right hand curve, x-rays only. Left hand curve, x-rays delivered starting 30 min after i.p. injection of 1 mg/g bodyweight of Ro-07-0582.

is the same as the ratio of TCD50s. This similarity provides clear evidence that the Ro-07-0582 actually reaches all the hypoxic cells and sensitizes them by the factor 1·8, and clear evidence against the alternative possibility that the drug reaches only some of the hypoxic cells. The corresponding slope ratio for the results on metronidazole previously published (Begg *et al.*, 1974) was, however, consistent with either theory.

An analysis was also done of delay in time to regrow to 8 mm. Because about half of the tumours were controlled, *i.e.* delay for them was infinite, the average of the reciprocal regrowth times for each dose group was plotted against x-ray dose. The enhancement ratio obtained graphically varied from 1·8 to 2, in good agreement with the ratio obtained from the TCD50 values.

The incidence of metastases did not appear to be influenced by Ro-07-0582. In mice whose tumours had been controlled for 150 days, 4 of 57 mice receiving both Ro-07-0582 and x-rays developed visible metastases, compared with 5 of 54 mice receiving x-rays only. The incidence of metastases in mice with locally recurrent tumours was 6/41 and 2/44 respectively (not significant difference, $\chi^2 = 1{\cdot}4$). The incidence of metastases was not influenced by metronidazole either in the previously described experiment (Begg *et al.*, 1974). Where tumours had been controlled for 150 days, 2/47 mice receiving x-rays and metronidazole developed metastases (compared with 1/20 in contemporaneous controls). The incidence for mice with locally recurrent tumours was 5/21 (cf. 4/14 in controls). These results are important in view of the finding that the drugs alone, without x-irradiation, caused a small delay in regrowth or reduction in size of the solid tumours (Begg *et al.*, 1974; Denekamp and Harris, unpublished). This delay or regression cannot be attributed to a drug stimulated loss of viable cells from the tumour volume.

When Ro-07-0582 was given 20 min *after* finishing the x-ray exposure, the TCD50 for 50% males and females (obtained from an experiment with 23 males and 25 females) was 4457 ± 220 rad (s.e.mean), which is not significantly different from the x-ray only group.

### DISCUSSION

The enhancement ratio ER = 1·82 means that, in this tumour system, at a dose of 3800 rad the proportion cured rises from 30% in control mice to nearly 100% in mice treated with 1 mg/g of Ro-07-0582 (Fig.). This value is similar to the ER of 1·9 found for the KHT sarcoma irradiated in air breathing mice and assayed *in vitro* (Rauth, unpublished); slightly less than the ER of 2·1 for regrowth delay in the murine carcinoma NT (Denekamp and Harris, unpublished); and of 2·1 for the CBA mouse sarcoma F irradiated *in vivo* and assayed *in vitro* by Dr N. J. McNally; and of about 2 found by Dr L. J. Peters using local control of intradermal squamous cell carcinomata; but somewhat greater than the ER of 1·6 observed by Mr A. C. Begg using either regrowth or loss of incorporated $^{125}$IUdR after treating the CBA mouse sarcoma F with x-ray doses of 1000–1500 rad, (private communications). The latter enhancement may have been made low by the use of the relatively low x-ray test dose. All the results quoted are for air breathing mice given 1 mg/g of Ro-07-0582 intraperitoneally 20–60 min before irradiation.

These results show that a large increase in the proportion of tumours locally controlled can be obtained using radiosensitizing drugs and x-rays. The drug appears to diffuse out from capillaries to the hypoxic cells normally present in tumours and is effective as a sensitizer of these hypoxic cells *in vivo*. The enhancement ratios, like those for other sensitizers tested *in vivo* (Denekamp and Michael, 1972; Hewitt and Blake, 1970; Sheldon and Smith, 1974), are somewhat less than would be expected from the *in vitro* results with concentrations in the medium of drug equal to the peak serum concentrations *in vivo*, although firm values for concentration of the drug in the relevant hypoxic cells are not known and would be difficult to obtain.

These ERs were obtained using relatively large quantities of drug: 1 mg/g corresponds to 50 g to a 50 kg patient on a weight-for-weight basis. This may correspond to 20–80 g on the basis of peak serum concentrations measured after very low doses. No such measurements in patients for Ro-07-0582 have yet been made at these high doses.

The main toxicological problem in the clinical use of metronidazole as a radio-sensitizing drug appears to be the acute nausea caused by several repeated doses of 5–10 g (*i.e.* 50–100 mg/kg) which persisted for 1 or 2 days after stopping the administration of the drug (Urtasun *et al.*, 1974; Deutsch *et al.*, 1974). Therefore, it is not expected that significantly higher doses than these can be used clinically. Ten to 30 repeated doses would be needed for a course of radiotherapy depending on whether 2, 3 or 5 sessions per week were administered, with 2 sessions per week obviously preferable on toxicological grounds.

An increase in the incidence of lymphosarcomata and lung tumours in mice, by approximately a factor of 2, has been reported for mice given daily doses of up to about 830 mg/kg of metronidazole throughout life (Rustia and Shubik, 1972). No such effect was found in rats (Cohen *et al.*, 1973). This is probably not a major hazard for patients who already have a malignant tumour requiring treatment.

A more serious question arises from reports of cerebellar damage in dogs. Repeated daily doses given to dogs of either metronidazole (150 or 250 mg/kg) or the 2-nitroimidazole compound Ro-07-1051, similar in structure to Ro-07-0582, (50, 100 or 150 mg/kg) caused bouts of ataxia, then continuous muscle spasm and cerebellar cell degeneration, ending in death. If the drug was withdrawn when the bouts began and sedatives were given, no further harmful effects were seen. Experiments in other species, however, indicate that the dog is atypically sensitive to this kind of cerebellar damage (Schärer, 1972). No such neurological symptoms were seen with similar or larger daily doses of Ro-07-1051 in mice, rats, guinea-pigs or rabbits. Further, the differences in structure and solubility of the two compounds suggest that Ro-07-0582 should not be more toxic than Ro-07-1051. The toxicology of Ro-07-0582 itself obviously has to be investigated further.

### CONCLUSIONS

The substantial enhancement ratio of 1·8 obtained for local control of the present solid tumour, using single doses of x-rays with 1 mg/g body weight of Ro-07-0582, compares well with the relative enhancement ratio of 1·7 for hypoxic cells obtained for fast neutrons.

The disadvantage is that the large drug doses (1000 mg/kg) required to obtain the high enhancement ratios which have been measured in murine tumours do not at present appear to be feasible clinically. At the lower drug doses which might be clinically usable (50–100 mg/kg) the sensitization enhancement ratios would be lower; they are being measured in a variety of experimental tumour systems and ERs in the range 1·0–1·5 can be expected (Rauth, unpublished; Hewitt *et al.*, personal communications). The effects of the drug when used with multiple small doses of x-rays are being tested. Different degrees of response in different types of tumour might be found and if so should be investigated further. Enhancement ratios exceeding 1·2 are required before an effect would be clinically detectable.

We thank Roche Products Ltd for supplies of Ro-07-0582, Dr G. E. Adams for his continued interest and encouragement, Dr J. Denekamp for constructive discussions, Drs E. H. Porter and L. J. Peters for the computer programme; Misses A. Marriott and J. Radmore for care of the mice and S. A. Hill for help with the experiment. We thank our colleagues in this laboratory, Drs J. Denekamp, H. B. Hewitt, N. J. McNally and L. J. Peters, and also Dr A. M. Rauth of the Ontario Cancer Institute, Toronto, for permission to quote their unpublished results.

Support for this work from the Cancer Research Campaign is gratefully acknowledged.

### REFERENCES

Adams, G. E. (1973) Chemical Radiosensitization of Hypoxic Cells. *Br. med. Bull.*, **29**, 48.

Asquith, J. C., Foster, J. L., Willson, R. L., Ings, R. & McFadzean, J. A. (1974*a*) Metronidazole (" Flagyl "). A Radiosensitizer of Hypoxic Cells. *Br. J. Radiol.*, **47**, 474.

Asquith, J. C., Watts, M. E., Patel, K., Smithen, C. E. & Adams, G. E. (1974*b*) Electron Affinic Radiosensitization: V. Radiosensitization of Hypoxic Bacteria and Mammalian Cells *in vitro* by some Nitroimidazoles and Nitropyrazoles. *Radiat. Res.* In the press.

Begg, A. C., Sheldon, P. W. & Foster, J. L. (1974) Demonstration of Hypoxic Cells in Solid Tumours by Metronidazole. *Br. J. Radiol.*, **47**.

Chapman, J. D., Reuvers, A. P. & Borsa, J. (1973) Effectiveness of Nitrofuran Derivatives in Sensitizing Hypoxic Mammalian Cells to X-rays. *Br. J. Radiol.*, **46**, 623.

Cohen, S. M., Erturk, E., von Esch, A. M., Crovetti, A. J. & Bryan, G. T. (1973) Carcinogenicity of 5-nitroimidazoles, 4-nitro-benzenes and Related Compounds. *J. natn. Cancer Inst.*, **51**, 403.

Denekamp, J. & Michael, B. D. (1972) Preferential Sensitization of Hypoxic Cells to Radiation *in vivo*. *Nature, New Biol.*, **239**, 21.

Denekamp, J., Michael, B. D. & Harris, S. R. (1974) Hypoxic Cell Radiosensitizers: Comparative Tests of some Electron Affinic Compounds using Epidermal Cell Survival *in vivo*. *Radiat. Res.* In the press.

Deutsch, G., Foster, J. L., McFadzean, J. A. & Parnell, M. (1974) Human Studies with High-dose Metronidazole, a Non-toxic Radiosensitizer of Hypoxic Cells. *Br. J. Cancer*, **31**. In the press.

Fowler, J. F. (1972) Current Aspects of Radiobiology as Applied to Radiotherapy. *Clin. Radiol.*, **23**, 257.

Fowler, J. F., Denekamp, J., Sheldon, P. W., Begg, A. C., Harris, S. R. & Page, A. L. (1974) Optimum Fractionation in X-ray Treatment of $C_3H$ Mouse Mammary Tumours. *Br. J. Radiol.* In the press.

Hewitt, H. B. & Blake, E. R. (1970) Studies of the Toxicity and Radiosensitizing Activity of Triacetoamine N-oxyl (TAN) in Mice. *Br. J. Radiol.*, **43**, 91.

Hill, R. P., Cheshire, P. J., Lindop, P. J. & Field, S. B. (1970) A Comparison of the Response of the Tumour and Normal Tissue in the Mouse Exposed to Single doses of Fast Neutrons or Electrons. *Br. J. Radiol.*, **43**, 894.

Rustia, M. & Shubik, P. (1972) Induction of Lung Tumors and Malignant Lymphomas in Mice by Metronidazole. *J. natn. Cancer Inst.*, **48**, 721.

Schärer, K. (1972) Selektive Purkinje-Zellschädigungen nach oraler Verabreichung grosser Dosen von Nitroimidazol-=Derivaten am Hund. *Verk. Deutsch. ges. Path.*, **56**, 407.

Sheldon, P. W. & Smith, A. M. (1975) Modest Radiosensitization of Solid Tumours in C3H Mice by the Hypoxic Cell Radiosensitizer NDPP. *Br. J. Cancer*, **31**. In the press.

Thomlinson, R. H. & Gray, L. H. (1955) The Histological Structure of some Human Lung Cancers and the Possible Implications for Radiotherapy. *Br. J. Cancer.*, **9**, 539.

Urtasun, R. C., Sturmwind, J., Rabin, H., Band, P. R. & Chapman, J. D. (1974) " High-dose " Metronidazole: a Preliminary Pharmacological Study Prior to its Investigational Use in Clinical Radiotherapy Trials. *Br. J. Radiol.*, **47**, 297.